# *Francisella tularensis* in the United States

**DOI:** 10.3201/eid1112.050728

**Published:** 2005-12

**Authors:** Jason Farlow, David M. Wagner, Meghan Dukerich, Miles Stanley, May Chu, Kristy Kubota, Jeannine Petersen, Paul Keim

**Affiliations:** * Northern Arizona University, Flagstaff, Arizona, USA; † Centers for Disease Control and Prevention, Fort Collins, Colorado, USA

**Keywords:** Arthropod vectors, disease reservoirs, Francisella tularensis, genetic diversity, North America, population distribution, tularemia, variable number tandem repeat loci, synopsis

## Abstract

The causative agent of tularemia, *Francisella tularensis*, is a formidable biologic agent that occurs naturally throughout North America. We examined genetic and spatial diversity patterns among 161 US *F. tularensis* isolates by using a 24-marker multiple-locus variable-number tandem repeat analysis (MLVA) system. MLVA identified 126 unique genotypes. Phylogenetic analyses showed patterns similar to recently reported global-scale analyses. We observed clustering by subspecies, low genetic diversity within *F. tularensis* subsp. *holarctica*, and division of *F. tularensis* subsp. *tularensis* into 2 distinct subpopulations: A.I. and A.II. The 2 *F. tularensis* subsp. *tularensis* subpopulations also represent geographically distinct groups; A.I. occurs primarily in the central United States, and A.II. occurs primarily in the western United States. These spatial distributions are correlated with geographic ranges of particular vectors, hosts of tularemia, and abiotic factors. These correlates provide testable hypotheses regarding ecologic factors associated with maintaining tularemia foci.

## Abstract 2

Subpopulations A.I and A.II. of *Francisella tularensis* subsp. *tularensis* are associated with unique biotic and abiotic factors that maintain disease foci.

Tularemia, also known as rabbit fever or deer-fly fever, is caused by the gram-negative intracellular pathogen *Francisella tularensis* (*1*). This bacterium was first identified in 1912 following reports of a plaguelike illness in ground squirrels in Tulare County, California (*2*). One of the most pathogenic microorganisms known, *F. tularensis* is currently listed as a category A select agent (*3*) because of its potential as a bioterrorism agent.

Since the discovery of this pathogen, 4 subspecies have been identified that exhibit distinct virulence and biochemical profiles as well as characteristic geographic distributions (*4*). Human disease is primarily associated with 2 *F. tularensis* subspecies: the highly virulent *F. tularensis* subsp. *tularensis* (type A), which is found only in North America, and the moderately virulent *F. tularensis* subsp. *holarctica* (type B), which is endemic throughout the Northern Hemisphere (*5*). Although *F. tularensis* subsp. *novicida* was recently reported in Australia, it is endemic primarily in North America and rarely isolated (*6*). *F. tularensis* subsp. *mediasiatica* is reported only from central Asian republics of the former Soviet Union (*7*).

Although the incidence of human tularemia is rare in the United States, the distribution of the pathogen appears ubiquitous (*8*). From 1981 to 1987, ≈60% of the cases reported in the United States occurred in Arkansas, Louisiana, Missouri, Oklahoma, or Texas (*9*). With the exception of localized outbreaks at Martha's Vineyard, Massachusetts, the central states of Arkansas, Missouri, Oklahoma, and South Dakota reported the highest incidence of the disease from 1990 to 2000 (*8*). Human tularemia incidence in the United States peaked in 1939 with 2,291 reported cases (*5*) and has since decreased to 100–200 cases annually (*8*).

In the United States, several blood-feeding arthropods serve as vectors for *F. tularensis*, including ticks (*Ixodidae*) and biting flies (*Tabanidae*) (*5*). Three ixodid tick species are important vectors in the United States: the American dog tick (*Dermacentor variabilis*), the Rocky Mountain wood tick (*D. andersoni*), and the Lone Star tick (*Amblyomma americanum*) (*5*). The deer fly (*Chrysops discalis*) was the first tularemia vector to be identified and is often associated with human disease in the western United States (*10**–**12*).

Tularemia infections have been documented in >200 species of mammals, as well as birds, reptiles, and fish (*4*). In North America, members of the family *Leporidae*, such as *Sylvilagus* spp. (cottontail rabbits) and *Lepus* spp. (hares), are important hosts (*5*). Despite these findings, the transmission cycle of *F. tularensis* is not well characterized because of the rare occurrence of natural outbreaks involving humans. As a result, ecologic and environmental factors promoting the maintenance of tularemia foci in North America remain largely unknown.

We recently identified a major division within *F. tularensis* subsp. *tularensis* (*13*). This division consists of the split between the highly diverse A.I. isolates, which include the SCHU S4 strain, and the less diverse A.II. isolates, which include the *F. tularensis* species type strain ATCC 6223 (*13*). Since this division was not previously recognized, no studies have yet explored ecologic factors that may serve as the basis for this structure.

In this study, we examined genetic-spatial patterns among North American *F. tularensis* isolates to better understand how geography may shape their genetic repertoire. In an attempt to identify factors that may influence the maintenance of endemic tularemia foci in the United States, we examined correlations between observed genetic groupings that were identified by using multiple-locus variable-number tandem repeat analysis (MLVA) and biotic and abiotic variables.

## Methods

### Isolates of *F. tularensis* and MLVA Subtyping

We examined 161 *F. tularensis* isolates, 158 from the United States and 3 from Canada. Subspecies analyzed included 83 *F. tularensis* subsp. *tularensis*, 72 *F. tularensis* subsp. *holarctica*, and 6 *F. tularensis* subsp. *novicida*. The originating laboratories for a subset of these isolates (n = 80) is reported elsewhere (*13*). All additional isolates were provided by the Centers for Disease Control and Prevention in Fort Collins, Colorado. A detailed description of the MLVA typing system and its use in examining phylogenetic relationships within *F. tularensis* are reported elsewhere (*13*).

### Phylogenetic, Spatial, and Statistical Analyses

A neighbor-joining dendrogram was generated by using PAUP (Sinauer Associates Inc., Sutherland, MA, USA). Distribution maps were generated with ArcView 3.3 (Environmental Systems Research Institute, Inc., Redlands, CA, USA); host and vector distributions were based on previously published data (*5**,**14**,**15*). Rank Mantel analyses were performed (*16*) by using PRIMER software (Primer-E, Ltd., Plymouth, UK). Genetic group (A.I. or A.II.) or location (California or not California) were used as the categoric factors for analysis of similarities (ANOSIM) (*17*). Spatial analyses were performed by using county centroid data from a subset of isolates with known county of origin. Within this subset, 1 representative was included from each set of isolates known to be from the same host or epidemiologically linked. Isolates examined included 49 *F. tularensis* subsp. *holarctica*, 30 *F. tularensis* subsp. *tularensis* subpopulation A.I., and 28 *F. tularensis* subsp. *tularensis* subpopulation A.II. A digital elevation model (Environmental Systems Research Institute, Inc.) was used to calculate mean elevation in each county of occurrence.

## Results

**Figure 1:**
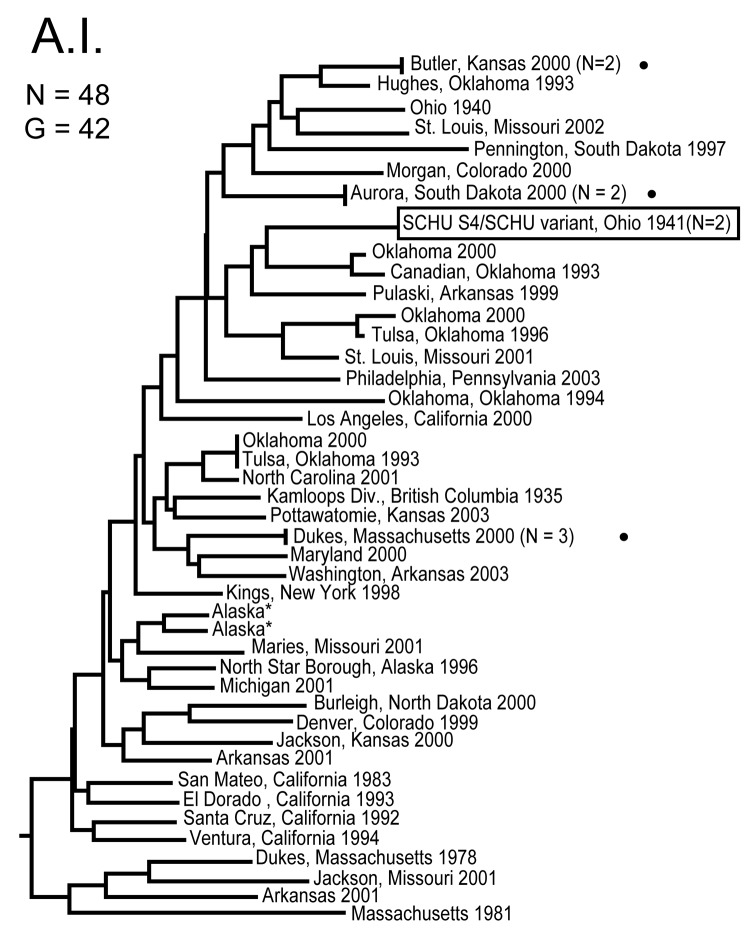
Genetic relationships among 48 North American Francisella tularensis subsp. tularensis A.I. subpopulation isolates based upon allelic differences at 24 variable number tandem repeat (VNTR) markers. County, state, and year of isolation are specified to the right of each branch or clade. G indicates number of distinct VNTR marker genotypes, dots indicate host-linked isolates, boxed designation indicates prominent F. tularensis subsp. tularensis laboratory strain SCHU S4, and asterisks indicate isolates with an unknown year of isolation.

**Figure 2:**
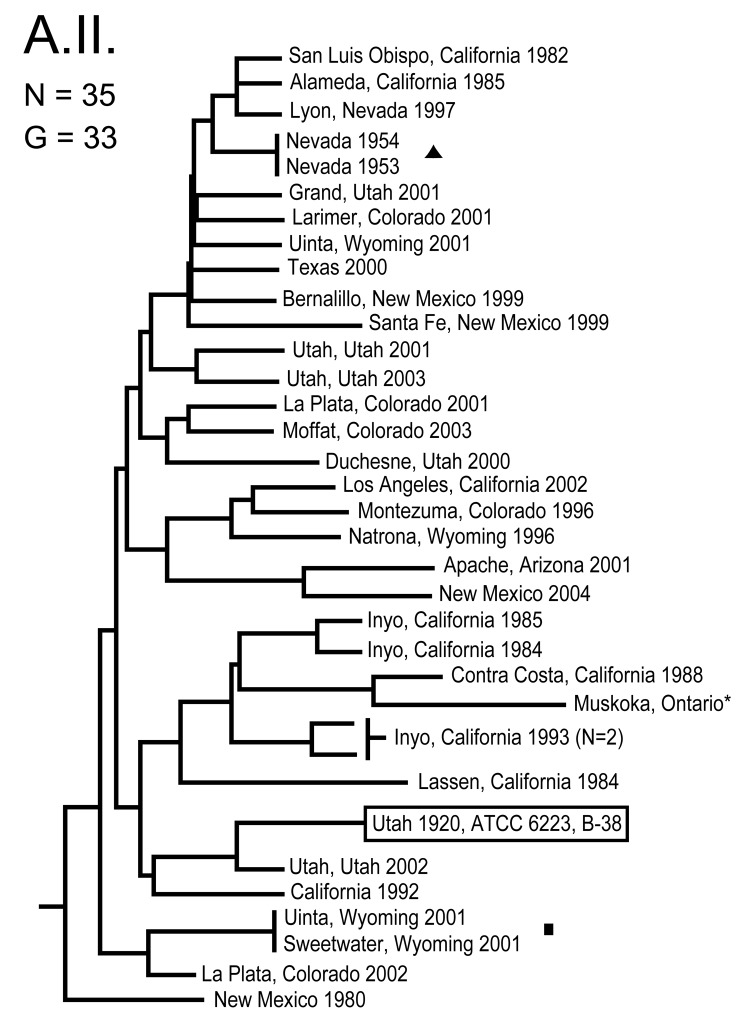
Genetic relationships among 35 North American Francisella tularensis subsp. tularensis A.II. subpopulation isolates based upon allelic differences at 24 variable number tandem repeat (VNTR) markers. County, state, and year of isolation are specified to the right of each branch or clade. G indicates number of distinct VNTR marker genotypes, triangle indicates epidemiologically linked isolate, asterisk indicates isolate with an unknown year of isolation, boxed designation indicates F. tularensis type strain B-38, and square indicates a set of genetically identical but epidemiologically unlinked isolates.

**Figure 3:**
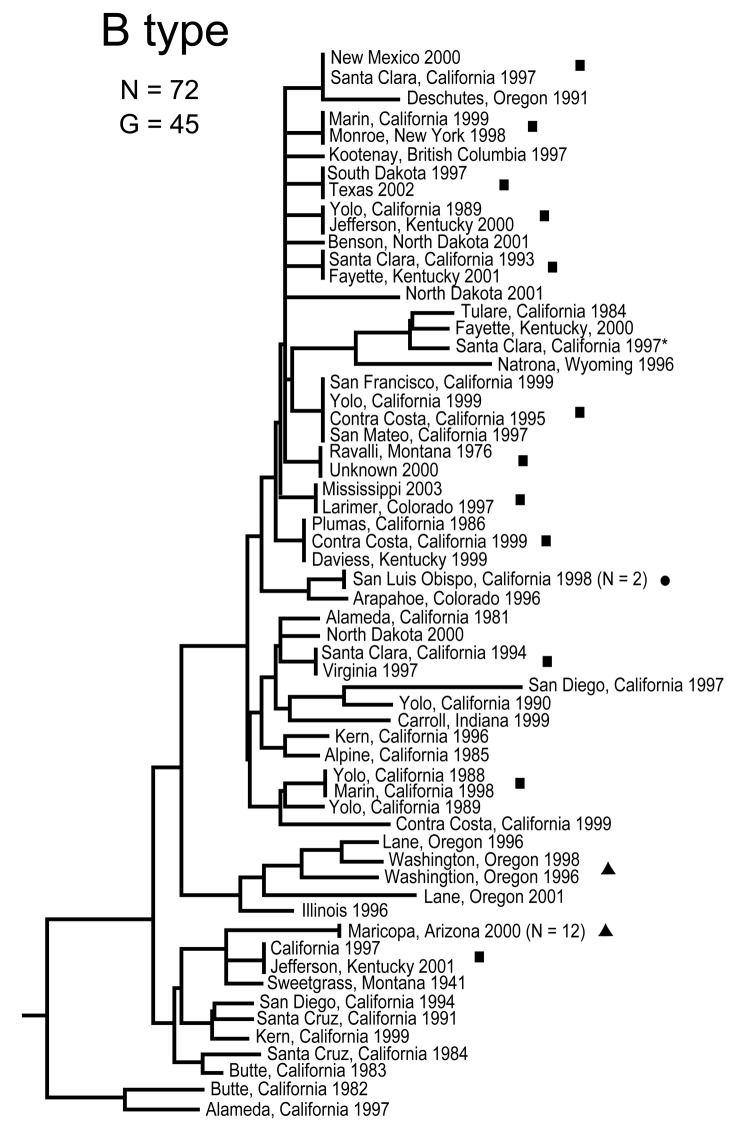
Genetic relationships among 72 North American Francisella tularensis holarctica B type isolates based upon allelic differences at 24 variable number tandem repeat (VNTR) markers. County, state, and year of isolation are specified to the right of each branch or clade. G indicates number of distinct VNTR marker genotypes, squares indicate genetically identical but epidemiologically unlinked isolates, asterisk indicates isolate with an unknown year of isolation, dot indicates a host-linked isolate, and triangles indicate epidemiologically linked isolates.

**Figure 4:**
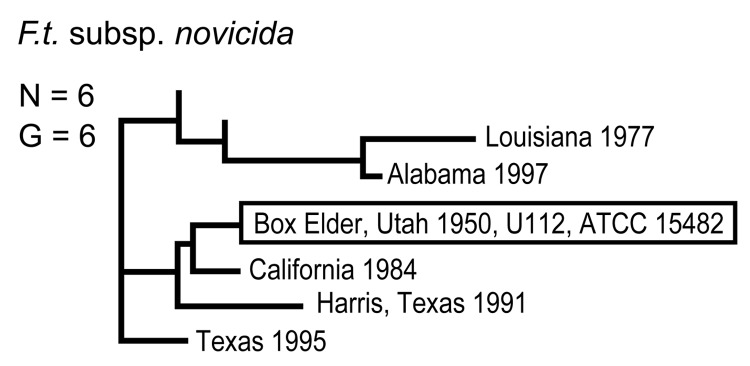
Genetic relationships among 6 North American Francisella tularensis subsp. novicida isolates based upon allelic differences at 24 variable number tandem repeat (VNTR) markers. County, state, and year of isolation are specified to the right of each branch or clade. G indicates number of distinct VNTR marker genotypes, and boxed designation indicates F. tularensis subsp. novicida type strain Utah 112 (U112).

**Figure 5:**
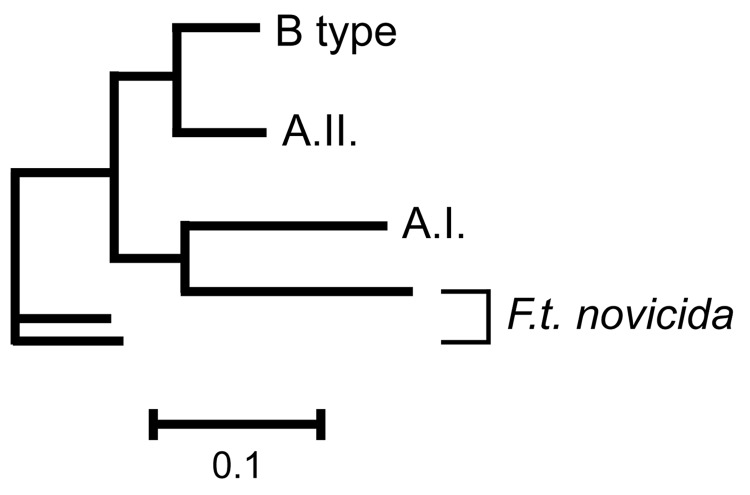
Phylogenetic relationships among subgroups A.I., A.II., B type, and Francisella tularensis subsp. novicida at 24 variable number tandem repeat markers. Scale bar represents genetic distance.

Neighbor-joining analysis of MLVA data identified 4 major genetic groups among the 161 North American *F. tularensis* isolates: *F. tularensis* subsp. *tularensis* subpopulation A.I., *F. tularensis* subsp. *tularensis* subpopulation A.II., *F. tularensis* subsp. *holarctica*, and *F. tularensis* subsp. *novicida* (Figures 1–5). The genetic groupings observed are consistent with the major genetics groups we described previously (*13*). In all cases, assignment of isolates to these genetic groups was consistent with their existing subspecies designations, which were based upon immunofluorescent, biochemical, and other molecular tests.

### Genetic Resolution

The MLVA typing system provided good genetic resolution (Figures 1–4). A total of 126 unique genotypes were observed among the 161 isolates. The average pairwise distance between isolates within the A.I. and A.II. subpopulations of *F. tularensis* subsp. *tularensis*, *F. tularensis* subsp. *holarctica*, and *F. tularensis* subsp. *novicida* was 0.324, 0.172, 0.144, and 0.310, respectively. MLVA provided complete discrimination among *F. tularensis* subsp. *tularensis* A.I. isolates, with the exception of 3 sets of isolates obtained from the same hosts (Figure 1). Among A.II. isolates, all but 2 sets of isolates were resolved by MLVA (Figure 2). Genetic resolution was poorest within *F. tularensis* subsp. *holarctica*; 14 sets of isolates were unresolved. Among these sets, only 2 were epidemiologically or host-linked, whereas many of the remaining 12 associated sets contain isolates from distant geographic locations (Figure 3).

### Geographic Distributions of Genetic Groups

**Figure 6:**
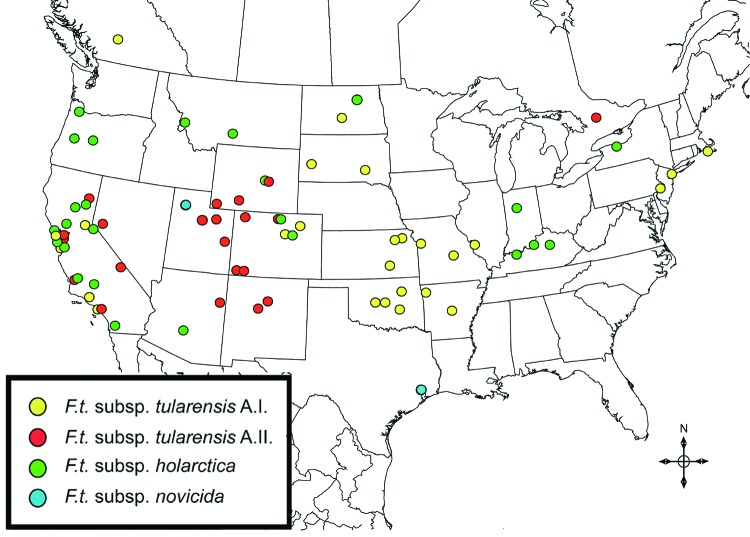
Spatial distribution of 125 Francisella tularensis isolates for which information on originating county was available. Locations (colored circles) correspond to county centroids. More than 1 subspecies was isolated from some counties in California (Alameda, Contra Costa, Los Angeles, San Luis Obispo, and Santa Cruz) and Wyoming (Natrona) (see Figures 1–3). In some cases, a single circle may represent instances where >1 sample of a given subspecies or genotypic group was isolated from a single county. Two isolates with county information, 1 from northern British Columbia and 1 from Alaska, are not shown.

The 4 genetic groups exhibited unique distributional patterns in geographic space (Figure 6). Isolates from *F. tularensis* subsp. *holarctica* were the most widespread, occurring in many of the lower 48 contiguous states, as well as British Columbia. With the exception of the 1920 Utah type strain (Utah 112) and 1 isolate from California, the other 4 *F. tularensis* subsp. *novicida* isolates were collected in southeastern states (Figures 4 and 6). The human incidence hotspot in the central United States (*8*) appears to be associated with the *F. tularensis* subsp. *tularensis* A.I. group (Figure 6). However, isolates from this group were also collected in Alaska, British Columbia, and California (Figures 1 and 6). In contrast, isolates of the *F. tularensis* subsp. *tularensis* A.II. subpopulation were collected primarily in the western United States, although some were also collected in Ontario and Texas (Figures 2 and 6). California is the only state that had isolates from all 4 genetic groups (Figures 1–4 and 6).

### Genetic-Geographic Correlations

Only within *F. tularensis* subsp. *tularensis* subpopulation A.II did genetic and geographic distances show a correlation (ρ = 0.340, p = 0.0009). No significant correlation was found between genetic and geographic distances within *F. tularensis* subsp. *tularensis* subpopulation A.I. (ρ = –0.009, p = 0.5239) or *F. tularensis* subsp. *holarctica* (ρ = 0.033, p = 0.3328).

### *F. tularensis* subsp. *tularensis* A.I. and A.II. Subpopulations

Based on ANOSIM, A.I. and A.II. isolates form 2 distinct groups in geographic space (R = 0.336, p<0.001). We found no evidence (R = –0.048, p = 0.639) that *F. tularensis* subsp. *tularensis* A.I. isolates from California (n = 5) are genetically distinct from A.I. isolates found in the other 47 contiguous states (n = 23).

**Figure 7:**
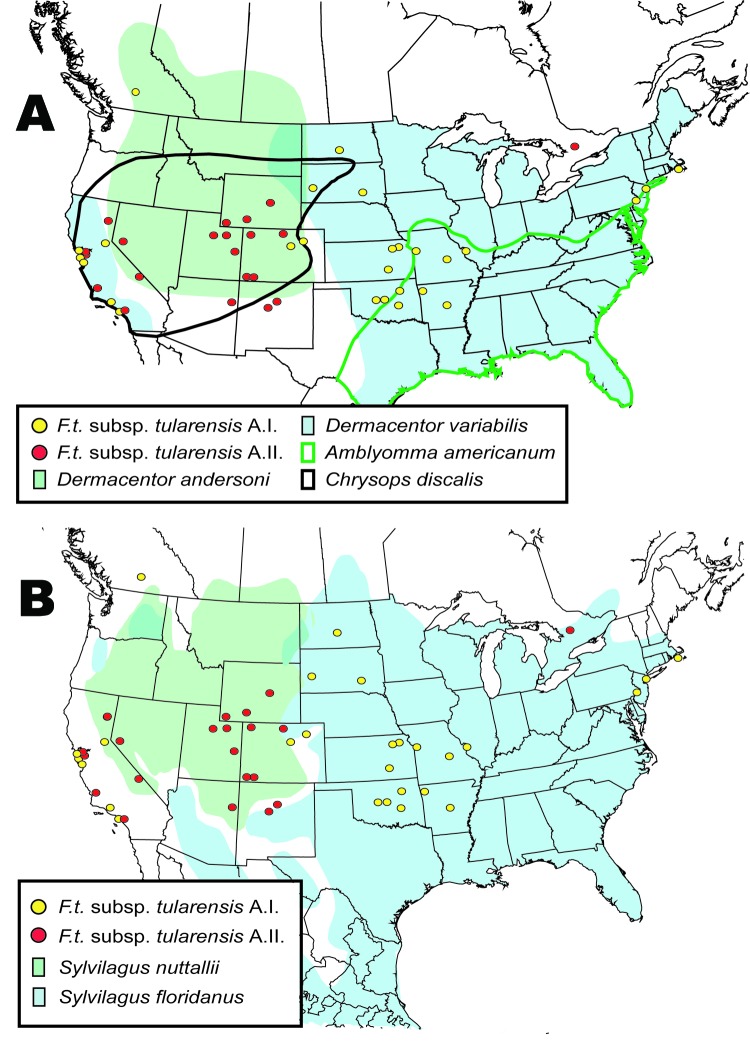
Spatial distributions of isolates from the A.I. and A.II. subpopulations of Francisella tularensis subsp. tularensis relative to A) distribution of tularemia vectors Dermacentor variabilis, D. andersoni, Amblyomma americanum, and Chrysops discalis; and B) distribution of tularemia hosts Sylvilagus nuttallii and S. floridanus.

The geographic distributions of the A.I. and A.II. subpopulations are associated with distinct abiotic and biotic factors, including known tularemia vectors and hosts. The mean elevation in counties where A.I. subpopulation genotypes were isolated was significantly lower (451.7 m, standard error [SE] 84.9; U = 211.5, p<0.001, by Mann-Whitney U test) than the mean elevation in counties where A.II. subpopulation genotypes were isolated (1,400.9 m, SE 175.2). The geographic distribution of A.I. isolates is closely associated with the distribution of the vectors *A. americanum* and *D. variabilis*; both *D. variabilis* and the A.I. isolates occur primarily in the central and eastern United States but also in California (Figure 7A). The main geographic cluster of A.II. isolates is associated with the distributions of 2 known tularemia vectors, *D. andersoni* and *C. discalis* (Figure 7A). Finally, the main geographic distributions of A.I. and A.II. isolates are each associated with the distributions of different rabbit hosts, *S. floridanus* and *S. nuttallii*, respectively (Figure 7B).

## Discussion

This study provides an ecogenetic analysis of *F. tularensis* in the United States and contributes new insights into this human health threat and potential biologic weapon. Our analyses categorized North American *F. tularensis* isolates into 4 previously recognized groups (13) and provided good genetic resolution within those groups (Figures 1–5). These findings indicate that MLVA is useful for examining continent-scale patterns of genetic diversity in *F. tularensis*. We focus here on a more detailed discussion of *F. tularensis* population structure on a continentwide scale and the ecologic correlates and associations of specific groups.

We observed relatively little genetic diversity within *F. tularensis* subsp. *holarctica* (Figure 3) despite analyzing samples from across North America (Figure 6). The genetic diversity that exists within this subspecies does not appear to be related to geographic distance. The lack of geographic differentiation, coupled with the low genetic diversity of *F. tularensis* subsp. *holarctica* in the United States, is consistent with rapid transmission of a recently emerged pathogen across great distances.

Unlike *F. tularensis* subsp. *holarctica*, the A.I. and A.II. subpopulations within *F. tularensis* subsp. *tularensis* are genetically distinct and geographically differentiated. The spatial distributions of these 2 subpopulations are associated with large differences in elevation, with A.I. occurring at lower elevations than A.II. Elevation alone is unlikely to influence the distribution of different groups within *F. tularensis* subsp. *tularensis*. We examined elevation because it is a single measurement that is highly correlated with other, more biologically relevant factors that may influence host and vector distributions, such as temperature, rainfall, and distribution of major vegetation types (18). The A.I. and A.II. subpopulations may have adapted to transmission and maintenance by specific vectors and hosts, leading to niche separation. This idea is supported by the striking association between the respective distributions of the A.I. and A.II. subpopulations and the distributions of specific tularemia vectors and hosts (Figure 7). Our results indicate that *S. floridanus* may be an important host for the A.I. subpopulation and *S. nuttallii* for the A.II. subpopulation (Figure 7B).

The A.I. and A.II. subpopulations within *F. tularensis* subsp. *tularensis* are associated with specific vector species, and movement of these vectors may have dispersed the pathogen across the United States. The distribution of the A.I. subpopulation is spatially correlated with *A. americanum* and the American dog tick *D. variabilis* (Figure 7A). The transport of dogs and, consequently, *F. tularensis*–infected *D. variabilis* may explain the lack of genetic-spatial correlation within this group, as well as the occurrence in California of both *D. variabilis* and the A.I. subpopulation of *F. tularensis* subsp. *tularensis*. Tularemia-infected *D. variabilis* could have been introduced into California through dogs during human westward migration in the 19th or 20th centuries. This hypothesis is consistent with the urban distribution of *D. variabilis* in California (*19*). Whatever the timing, A.I. isolates from California do not form a genetic group that is distinct from other A.I. isolates, which is suggestive of multiple introductions to California from the eastern United States. In contrast, the information in Figure 7 suggests the primary focus of the *F. tularensis* subsp. *tularensis* A.II. subpopulation is in the western United States and that this focus is associated with the vectors *D. andersoni* and *C. discalis*.

**Figure 8:**
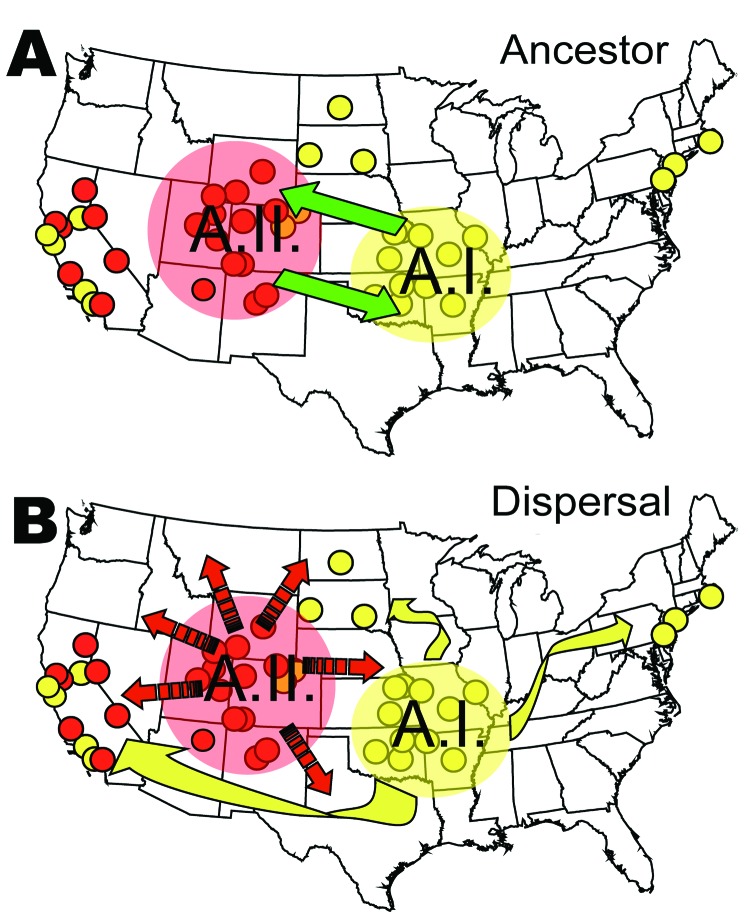
Genetic and spatial data of the A.I and A.II subpopulations of Francisella tularensis subsp. tularensis in the United States. A) Ancestral status of these 2 subpopulations is unclear; either could have founded the other, or a third unknown subpopulation could have been the ancestor. B) Highly restricted bacterial-endemic regions could now be breaking down because of human-mediated dispersal of the pathogen across the country. The small circles indicate the spatial distribution of the A.I and A.II isolates, as shown in Figure 6.

The evolutionary linkage of the A.I. and A.II. subpopulations within *F. tularensis* subsp. *tularensis* may be ancient (Figure 8A). Large MLVA distances separate these types (*13*) and are equivalent to those separating other *F. tularensis* subspecies (Figure 5). The current spatial distribution and genetic distances distinguishing the A.I. and A.II. subpopulations may have been shaped by Pleistocene refugia. The greater diversity observed in the A.I. subpopulation is consistent with an older age, more rapid evolution in this focus, or a historical genetic bottleneck unique to the A.II. subpopulation that occurred after A.I.–A.II. separation. Evolutionary rates are accelerated in certain ecologic scenarios and retarded in others. However, if equal evolutionary rates between the A.I. and A.II. subpopulations are assumed, A.I. is older and may have been the founding population for A.II. More robust phylogenetic analysis that uses slowly evolving characters (*20**,**21*) should eventually root this relationship.

The lower Midwest tularemia focus (*8*) may have been a dispersal source for other A.I. populations in the United States. In this model (Figure 8B), continentwide dispersal may have occurred as recently as the advent of modern transportation (e.g., rail or automobile traffic). A locally robust population of *F. tularensis* subsp. *tularensis* A.I. may have been relatively isolated until European colonists dispersed this pathogen throughout the continent. The rapid and long-range dispersal of infected animals or vectors would be similar to an evolutionary radiation with little correlation to spatial parameters.

Such rapid dispersal also may be a function of the recent introduction of lagomorph species into these areas. In the first half of the 20th century, hundreds of thousands of rabbits and hares were shipped from central states to eastern states (*5**,**22*), and some of these shipments included carcasses infected with *F. tularensis* (*23*). Before 1937, no cases of tularemia were reported from Massachusetts (*5*). These reports suggest that mass introductions of cottontail rabbits for sporting purposes ultimately may have helped shape the geographic distribution of this pathogen in the United States. Clearly, this anthropogenic factor played some role in dispersing the pathogen from the central regions of the United States to eastern regions where tularemia is now endemic.

The overall incidence of human tularemia infections in the United States appears to arise from areas where we showed the prevalence of the A.I. subpopulation of *F. tularensis* subsp. *tularensis*. Some of the main human incidence hotspots in the United States, Arkansas, Kansas, Massachusetts, Missouri, Oklahoma, and South Dakota (*8*), are all associated with A.I. (Figures 1 and 6). This distribution may be the result of a successful group within the *F. tularensis* subsp. *tularensis* A.I. subpopulation or favorable ecologic conditions that promote disease maintenance and transmission in this region.

## Conclusions

Our results confirm the presence of 2 distinct subpopulations within *F. tularensis* subsp. *tularensis* and indicate that these groups are geographically distinct and associated with unique biotic and abiotic factors. These findings are important because *F. tularensis* subsp. *tularensis* is most often associated with human tularemia in the United States. The ecologic correlates identified here provide a framework for developing testable hypotheses regarding niche separation between the A.I. and A.II. subpopulations and should inform future studies addressing the transmission dynamics and persistence of *F. tularensis* in North America.
